# Nanotechnology in Stroke: New Trails with Smaller Scales

**DOI:** 10.3390/biomedicines11030780

**Published:** 2023-03-04

**Authors:** Karlo Toljan, Anushruti Ashok, Vinod Labhasetwar, M. Shazam Hussain

**Affiliations:** 1 Department of Neurology, Neurological Institute, Cleveland Clinic, Cleveland, OH 44195, USA; 2 Biomedical Engineering, Lerner Research Institute, Cleveland Clinic, Cleveland, OH 44195, USA; 3 Cerebrovascular Center, Department of Neurology, Neurological Institute, Cleveland Clinic, Cleveland, OH 44195, USA

**Keywords:** nanoparticles, drug delivery, thrombolysis, drug targeting, reperfusion injury, oxidative stress

## Abstract

Stroke is a leading cause of death, long-term disability, and socioeconomic costs, highlighting the urgent need for effective treatment. During acute phase, intravenous administration of recombinant tissue plasminogen activator (tPA), a thrombolytic agent, and endovascular thrombectomy (EVT), a mechanical intervention to retrieve clots, are the only FDA-approved treatments to re-establish cerebral blood flow. Due to a short therapeutic time window and high potential risk of cerebral hemorrhage, a limited number of acute stroke patients benefit from tPA treatment. EVT can be performed within an extended time window, but such intervention is performed only in patients with occlusion in a larger, anatomically more proximal vasculature and is carried out at specialty centers. Regardless of the method, in case of successful recanalization, ischemia-reperfusion injury represents an additional challenge. Further, tPA disrupts the blood-brain barrier integrity and is neurotoxic, aggravating reperfusion injury. Nanoparticle-based approaches have the potential to circumvent some of the above issues and develop a thrombolytic agent that can be administered safely beyond the time window for tPA treatment. Different attributes of nanoparticles are also being explored to develop a multifunctional thrombolytic agent that, in addition to a thrombolytic agent, can contain therapeutics such as an anti-inflammatory, antioxidant, neuro/vasoprotective, or imaging agent, i.e., a theragnostic agent. The focus of this review is to highlight these advances as they relate to cerebrovascular conditions to improve clinical outcomes in stroke patients.

## 1. Introduction

**Figure 1:**
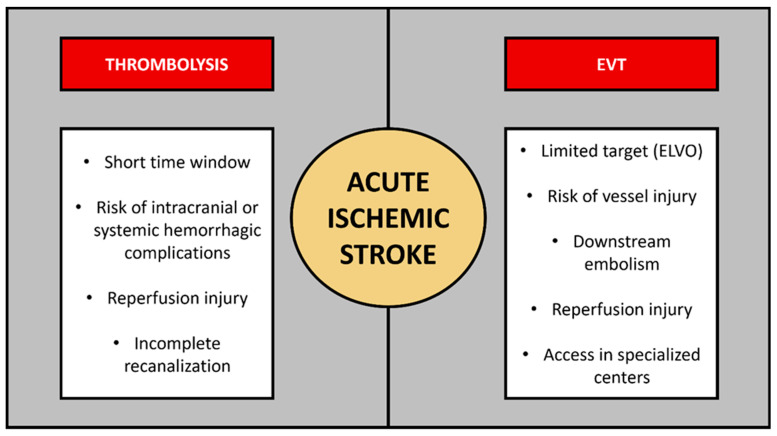
Current acute ischemic stroke specific therapies and challenges associated with each modality. In addition to limited eligibility for acute ischemic stroke treatments, specific challenges exist in case of thrombolysis or thrombectomy, respectively. ELVO—emergent large vessel occlusion; EVT—endovascular thrombectomy.

Globally, stroke is a leading cause of death and long-term disability [1]. In the United States, it is the 5th leading cause of death, with 795,000 new cases per year [2]. The estimated cost to the healthcare system is $28 billion per year, with another $17.5 billion as an indirect cost due to lost productivity or mortality [3]. Ischemic stroke caused by arterial occlusion is responsible for the majority of strokes. Current acute ischemic stroke treatment is based on the early application of intravenous thrombolytics [4], primarily alteplase (recombinant tissue plasminogen activator, tPA), and in a selected patient population, endovascular thrombectomy (EVT) [5]. Although both modalities have enabled effective treatment of acute ischemic stroke, limitations are still present (Figure 1). These treatments were initially utilized in <5% of acute ischemic stroke cases by the early 2010s [5,6,7], but with growing evidence and updated guidelines, the theoretic eligibility for such presentations at a comprehensive stroke center increased to ~20% for EVT and ~35% for thrombolysis [8,9]. Current guidelines support the use of alteplase within a 4.5 h period since symptom onset and EVT use in the first 24 h period, based on the appropriate selection of the patient population [4].

**Figure 2:**
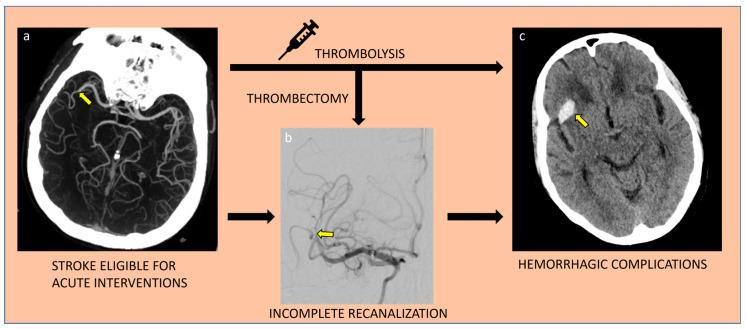
Major challenges of current acute ischemic stroke recanalizing treatments; incomplete recanalization and hemorrhagic complications. Yellow arrow respectively points to the site of vessel occlusion (**a**), incompletely recanalized vessel during intervention (**b**), and hemorrhagic transformation of the initial ischemic infarct territory (**c**).

Developing a thrombolytic agent with an extended window for stroke treatment is an unmet clinical need, and is an active area of research, with a potential benefit extending to selected cases based on salvageable penumbral tissue as determined by neuroimaging assessing perfusion or diffusion. In addition, there is a significant fraction of patients with “wake-up stroke” (patients who go to sleep but are awakened with stroke symptoms, representing roughly one in five acute ischemic strokes) [10]. Besides time as the main limiting factor for acute stroke treatment, reperfusion injury [11] and hemorrhagic complications are the most prominent life-threatening side effects once recanalizing therapies have been applied (Figure 2) [12,13]. In the subset of stroke with emergent large vessel occlusion (ELVO), tPA is less effective due to its limited contact and penetration within a large clot to cause thrombolysis and recanalization due to its short half-life (~5 min) [14,15,16].

EVT has emerged as the standard of care for patients with ELVO, but its effectiveness is also time dependent [17,18] and it must be performed by trained neurointerventionalists, requiring patients to be directed to highly specialized centers [19]. The procedure is associated with a high risk of vascular damage/perforation if not performed properly. Often, and depending on the clot composition, particularly if it is a fibrin-rich hard clot, multiple passages during EVT are required to remove the it. With each passage, there is increased risk of vascular damage [20]. In addition, thrombus fragments formed as a result of the procedure can obstruct distal and small vasculature, which may require the use of tPA [21]. In general, ELVO remains a challenge in cases of thrombolysis only, whereas application of a thrombolytic agent with successful thrombectomy has a similar risk of hemorrhagic complications as compared to thrombolysis alone (2–7%) [22,23,24].

### 1.1. Effect of Clot Composition on Thrombolysis

Composition, features, and location of the clot impact the effectiveness of therapies. Longer clots and clots in large vessels [25], such as a distal internal carotid artery or basilar artery, are associated with lower rates of recanalization following the use of tPA only [26,27], rather than tPA with EVT combination [28,29,30,31]. Establishing stroke etiology may discern whether occlusion occurred as a likely isolated acute process (e.g., cardioembolism) or a combination of acute and chronic processes (e.g., embolism of an atherosclerotic vessel), which also has implications regarding clot composition and expected acute treatment response [32].

Red blood cell-rich clots, suggestive of an earlier phase of thrombosis and cardioembolism as etiology, respond better to recanalizing attempts with tPA than the fibrin and platelet-rich clots [17,18]. However, after a fresh clot is formed, granulocyte infiltration is noted (lytic thrombus), followed by smooth muscle cell ingrowth (organized thrombus), implying decreased specificity of thrombus type as it ages [33]. In the acute stroke population, there is also a freshly formed component on top of any underlying subacute or chronic thrombus [34]. Platelet-rich clots, associated with large artery atherosclerosis as the etiology [34] are more resistant to tPA treatment than red blood cell-rich clots associated with cardioembolism [32] as they appear to be less permeable to thrombolytic agent [35,36,37,38].

Recently, there has been a renewed interest in the use of a 3rd generation thrombolytic, tenecteplase, which is more fibrin specific and more resistant to plasminogen activator inhibitor-1 with a longer plasma half-life [39] which may offer better recanalization rates for ELVO as compared to the currently used alteplase (half-life = 5 vs. 17 min) [40]. In addition, because of the longer half-life, tenecteplase can be administered as a single bolus injection, whereas tPA is administered first as a 10% bolus dose followed by a slow infusion over a period of 60 min [41]. The slow infusion of the second part of the dose is to sustain the effect of tPA so that there is more contact time with the clot to cause its lysis and also to reduce the risk of non-specific hemorrhage that may occur by giving the entire dose as a bolus injection [42]. Although approved for acute myocardial infarction, recent data indicate that tenecteplase is just as good as tPA, with no additional risk, and in some cases better regarding the functional outcome at 90 days, outcomes of post-thrombolytic bleeding, and recanalization/reperfusion rates following thrombectomy [43].

### 1.2. Reperfusion and Hemorrhagic Injury

Hemorrhagic complications remain the major concern when applying reperfusion therapies or EVT in the setting of acute ischemic stroke [44]. Overall, the risk of hemorrhage from thrombolysis with or without EVT is approximately 6%, with increased risk seen in those treated later in the therapeutic time window [45]. Larger stroke size, older age, high systolic blood pressure, or concurrent use of antiplatelets are additional major risk factors associated with hemorrhagic transformation following acute ischemic stroke treatment [45]. It is theorized that hemorrhagic risk is mainly driven by recanalization and reperfusion injury [46,47,48], though tPA itself may lead to tissue injury in areas of infarcted tissue with already compromised blood-brain barrier (BBB) integrity [49].

**Figure 3:**
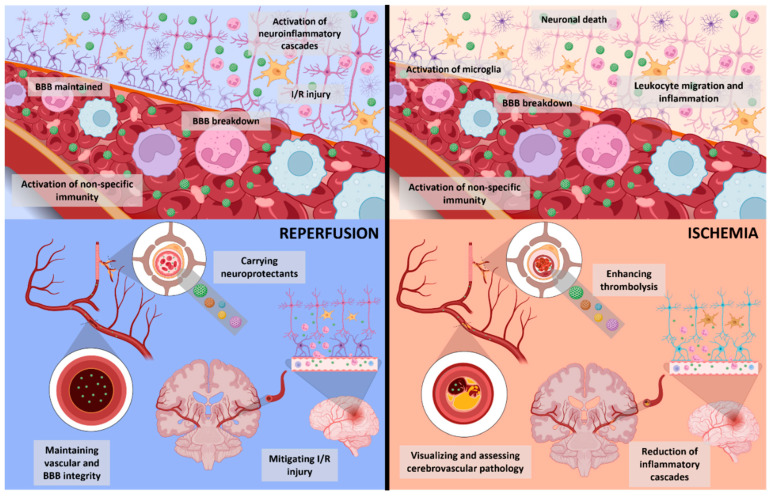
Schematic depicting acute changes with reperfusion following successful vessel recanalization (on the **left**) contrasted with ischemia following vessel occlusion (on the **right**). Despite vessel recanalization, there may still be disruption in the function or structure of BBB or ischemic changes, compounded by neuroinflammatory cascades triggered by reperfusion or direct effects of thrombolytics. Nanoparticles and nanoconjugates (green dots) may help target detrimental pathophysiological changes in reperfusion and ischemia by delivery of neuroprotective and vasoprotective agents to minimize vessel injury, or by ameliorating ischemia-triggered cascades, and neuroinflammation. Theragnostics hold the potential to improve diagnostic and prognostic tools and bolster current acute treatments. BBB—blood-brain barrier, I/R—ischemia/reperfusion.

Acute stroke is associated with local and systemic inflammatory response [50] with activation of non-specific immunity, including neutrophils [51] and complement system [52] as well as platelets (Figure 3) [53]. Such events are associated with rapid production of reactive oxygen and nitrogen species (ROS; RNS) and secondary damaging effects encompassing mitochondria, endothelial cells, and, in case of loss of BBB integrity, even neurons and glial cells [50,54]. Consequently, there is a higher expression of endothelial adhesion molecules and leukocyte recruitment, secondary cascades producing damage-associated molecular patterns [55], and further generation of ROS and RNS as a pathophysiological vicious cycle [56].

In the case of successful reperfusion, classic features of ischemia/reperfusion (I/R) injury are present (Figure 3) [46,57]. Notably, those are represented by increased ROS and RNS formation as a non-specific inflammatory response, including the innate immune system and mitochondria. Additional oxidative cascades are mediated by xanthine oxidase as a result of functional enzyme modification due to ischemia or activation of nicotinamide adenine dinucleotide phosphate oxidase as part of oxidative stress [57,58]. Moreover, the restored circulation supplying more oxygen to the area of completed infarct or penumbral tissue, possibly already affected by considerable oxidative stress [59], may induce or worsen neuroinflammation and associated excitotoxicity, as well as vasogenic edema or cytotoxic edema [46,57] (Figure 3). Although the aforementioned pathophysiological consequences are part of the same process, an individualized timeline is present, as demonstrated by clinical scenarios in which BBB integrity may be maintained even several days from initial stroke symptom onset [60].

Thus, there is an unmet clinical need to develop a thrombolytic agent which would reduce the risk of cerebrovascular complications, particularly hemorrhagic, ideally with an extended time window for treatment. Conversely, earlier availability of such improved treatment modalities should also reduce individual risks for reperfusion injury and its consequences. The other challenge to resolve is an agent that would be effective on the clots of different compositions (red clot vs. white clot). In this regard, nanotechnology and other mechanical approaches are being investigated to overcome some of the critical issues associated with the recanalization of cerebrovascular occlusions.

## 2. Nanoparticles and Nanoconjugates

**Figure 4:**
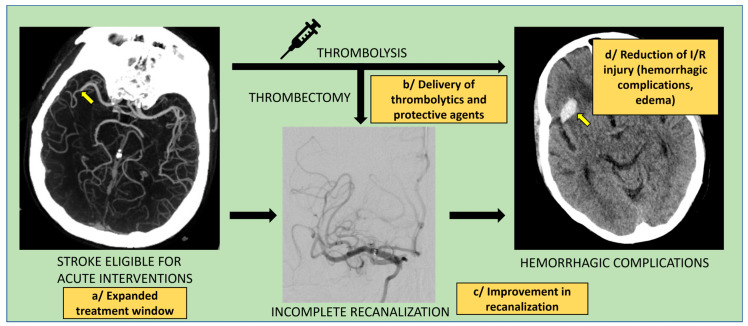
Main objectives for clinical application of nanoparticles in acute ischemic stroke. Nanoparticle-based approaches could improve acute ischemic stroke treatment by: expanding the treatment window eligibility (safer thrombolytics) (**a**), improving delivery modes for thrombolytics (increased affinity for thrombus, longer half-life) and enabling delivery of neuro/vaso-protective agents (**b**), enhancing recanalization rates with pharmacological, physical, or chemical function (**c**), and reducing ischemia/reperfusion (I/R) injury with subsequent decrease in infarct site complications (hemorrhagic transformation, tissue edema) (**d**).

A novel paradigm based on nanoparticles and nanoconjugates for the delivery of therapeutics and targeting is emerging for cerebrovascular diseases. For developing effective treatment for ELVO, different approaches have been explored to minimize the damage due to I/R injury. The injury is caused by a sudden resumption of blood flow, which is further aggravated with the presence of a thrombolytic agent such as tPA due to the risk of hemorrhagic complications [61]. In recent advances, a thrombolytic agent is either encapsulated or conjugated to the nanoparticle surface to enhance its efficacy. The main objectives of these efforts are to (a) reduce the risk of hemorrhage, (b) achieve better recanalization than with the currently used thrombolytic agents, (c) protect the cerebrovascular tissue from I/R injury, and (d) extend the window for treatment (Figure 4). A safer and more effective thrombolytic agent will benefit more acute stroke patients than those treated with the currently used thrombolytic agents.

### 2.1. Nanoparticles

Nanoparticles are submicron-sized particles, typically in the size range of 50 to 300 nm in diameter, particularly when used for drug delivery applications. Nanoparticles are mostly spherical in shape, but they have been synthesized with different architectures [62]. Depending upon their applications, nanoparticles are formulated using different biomaterials such as synthetic or natural polymers [63], lipids (e.g., glycerides, fatty acids, fatty alcohols, etc.), or metals (e.g., gold, iron oxide, etc.) [64,65]. Polymers could be synthetic (e.g., poly lactic-co-glycolic acid, PLGA; polylactide, PLL; poly-methyl methacrylate, PMMA, etc.) or natural (e.g., gelatin, chitosan, etc.) [63]. Nanoparticles such as cyclodextrins which contain a cargo to encapsulate therapeutic agents [66] or self-assembling polymer complexes, commonly referred to as micelles, can solubilize water-insoluble therapeutics [67]. Depending upon their specific applications, nanoparticles can be surface functionalized, such as with polyethylene glycol (PEG), commonly referred to as PEGylated nanoparticles that are stealth to the immune system and hence have longer circulation time than non-PEGylated nanoparticles [68]. In addition, PEG is used for conjugating nanoparticles to a targeting ligand [69].

### 2.2. Nanoparticles for Stroke Therapy

Although nanoparticles have been extensively investigated for drug delivery and imaging applications in other disease conditions, particularly for cancer therapies and for improving oral bioavailability of therapeutics, and in other emerging areas such as the ophthalmic delivery, for vaccine delivery, e.g., mRNA-based COVID-19 vaccine [70], their potential for cerebrovascular conditions has not yet been fully explored [71]. Depending upon the target or the objective, the size of nanoparticles is an important consideration [72]. Very small-sized nanoparticles (<10 nm) can rapidly clear through glomerular filtration and will be excreted [73], whereas too large-sized nanoparticles could impede their transport into the clot, carrying thrombolytic agents, or their transport of neuroprotective agents to the penumbra [74]. Nanoparticles have been evaluated for delivering therapeutic agents of different types—small molecular therapeutics and macromolecules. Therapeutic agent(s) of different types or pharmacological effects can be encapsulated or conjugated/adsorbed onto the surface [64,75]. Advantages of nanoparticle-based drug-delivery systems are multiple such as sustained/controlled release of the encapsulated agent, protection of unstable therapeutics, targeting, and/or altering biodistribution to limit unwanted side-effects [76].

**Figure 5:**
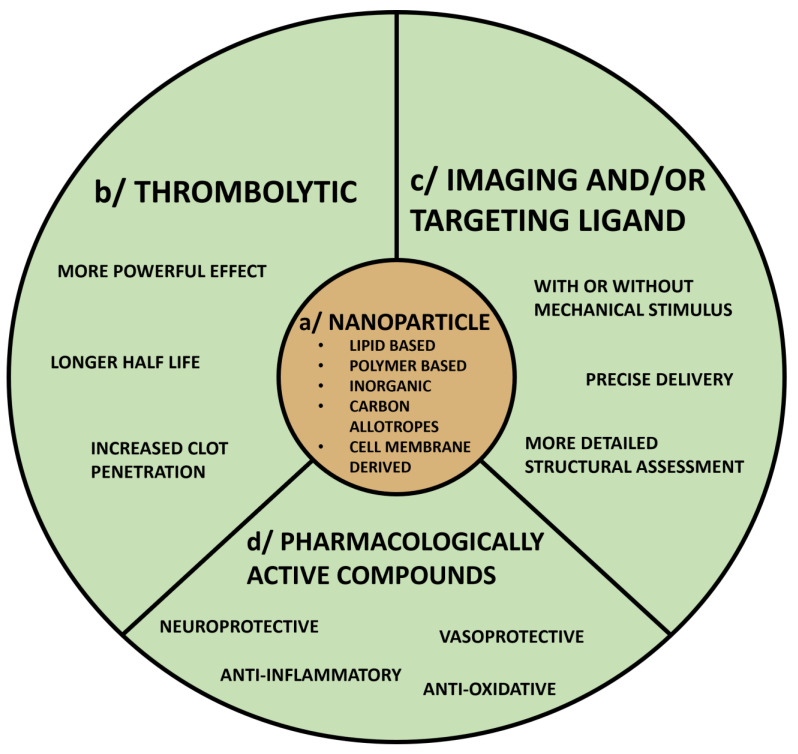
Nanoparticle-based formulations as tools in stroke diagnosis and treatment. Nanoparticle-based formulations have multiple potential applications for clinical management of acute ischemic stroke including delivery of thrombolytics and neuro/vaso-protective agents, improvement of imaging capabilities, and more precise targeting, which should enhance the safety profile of acute treatments. Key components of such formulations are nanoparticles (**a**), combined with either thrombolytics (**b**) and/or imaging or targeting ligands (**c**), or other components such as pharmacologically active compounds (**d**), which could decrease tissue injury and enhance recovery.

For this review, we refer to nanoconjugates as the formulation where the nanoparticle surface is modified. Nanoparticles, as such, can have either encapsulated therapeutics (within the polymer), conjugated (onto the surface), or have both encapsulated and conjugated. The surface can be modified with a targeting ligand. An attempt has been made to develop multifunctional nanoconjugates that would include complementary features to enable multi-target action for improved stroke therapy. With nanoparticles as a base, one could encapsulate a variety of agents that could be neuro/vascular protective and targeted toward the diseased vessel or specific component of the thrombus. Therefore, nanoparticle-based formulations that are being developed and investigated for ischemic stroke therapy are typically composed of three major components, (i) nanoparticle, (ii) thrombolytic agent such as tPA, and/or (iii) imaging agent/cerebrovascular-neuroprotective agent/targeting ligand (Figure 5). The following section will briefly discuss these components and some recent advancements in nanoconjugate-based drug-delivery systems relevant to the stroke condition.

#### 2.2.1. Nanoparticles with Thrombolytic Agents

Nanoparticles are investigated to deliver tPA or other thrombolytics that are either encapsulated or conjugated to nanoparticles. The advantage of encapsulation is that it temporarily suppresses the tPA activity while in the blood circulation, thus extending its half-life, allowing more contact time with the clot than tPA administered as a solution, therefore achieving better thrombolysis and also minimizing the risk of non-specific bleeding, due to controlled release of tPA [77,78]. In addition, encapsulation protects the thrombolytic agent from endogenous inhibitors of fibrinolysis (e.g., α-2-antiplasmin, α-2-macroglobuline, anti-C1 esterase, α-1 antitrypsin, and plasminogen activator inhibitor 1), thus potentiating the thrombolysis effect of the encapsulated thrombolytic agent [79]. Sustained release of the encapsulated agent also reduces the potential risk of tPA-mediated hemorrhage at non-target regions [80].

Immobilization/conjugation of tPA to the nanoparticle surface through covalent attachment offers more stability than free tPA due to their fixed conformation, which permits tPA to act directly on the thrombus. In this regard, multiple different types of nanoparticles with encapsulated or conjugated tPA and other thrombolytic agents have been formulated, such as PEGylated liposomes [81], silica-coated magnetic nanoparticle (SiO2-MNP) [82], magnetic nanoparticles, [83,84], gold nanoparticles (tPA/AuNP) [85], and polymeric nano-constructs (tPA-DPNs) [86].

#### 2.2.2. Nanoparticles with Imaging Agents

With the additional feature of imaging capability, one would be able to determine the localization of the nanoconjugate to the target vessel/thrombus, retention, and whether recanalization has occurred or not, which would help to detect/image the severity of stroke injury and determine the effective dose or dosing frequency of the nanoconjugate required for recanalization. Imaging techniques are used, including nanoparticle-based, to enable the diagnosis of cerebral vasculature pathologies, infarct region, compromised BBB integrity, and detect neuroinflammation markers [87]. Those techniques could be used in predicting complications such as massive cerebral edema and hemorrhagic transformation in the acute ischemic stroke [88].

Nanoparticles with a thrombolytic agent, but also with imaging features, have been developed and evaluated. For example, nanosized erythrocyte ghosts, which have a long circulation time, contained encapsulated indocyanine green (ICG) with tPA conjugated to the surface with fluorescence imaging and thrombolysis properties [89]. Similarly, bovine serum albumin (BSA) containing Mn and ICG as a contrast agent (MnCO_3_@BSA-ICG nanoconjugate) that comprises the magnetic resonance/photoacoustic (MR/PA) dual-modal imaging system has been explored to detect infarct area [90]. Biocompatible BSA–MnO_2_ nanoparticles [91], PEGylated superparamagnetic iron oxide nanoparticles (SPION) [92], and gadolinium (Gd)-based [93] contrast agents have been successfully synthesized for quantitative BBB permeability imaging.

Apart from a thrombolytic agent, other applications of nanoparticles are useful in stroke conditions. For example, fluorescent mesoporous silica coated SPION have been used to track neural progenitor cells migrating to the lesion site [94]. Gd-based contrast agent, (nanoGd conjugate), which can be internalized by phagocytic cells, has been explored to monitor neuroinflammation at the subacute stage of ischemic stroke [95]. Similarly, carbohydrate-functionalized core-shell silica magnetic nanoparticles which specifically bind to the endothelial transmembrane inflammatory proteins (E and P selectin) were tested to monitor post-stroke inflammation [96]. Due to the ability of mesenchymal stem cells (MSC) to migrate toward the neuro-inflammatory signal, MSC-derived exosome nanovesicles combined with gold nanoparticles were tested to analyze brain pathology in the stroke [97].

#### 2.2.3. Nanoparticles with Cerebrovascular-Neuroprotective Agents

Nanoparticles with thrombolytic properties containing neuroprotective agents against cellular and molecular markers involved in the secondary pathological mechanisms in stroke are developed to rescue hypoperfused penumbra from cell death [98]. Considering the role of platelets during thrombus formation, bioengineered nanoplatelet conjugated with neuroprotectant (ZL006e) for targeted delivery of tPA for ischemic stroke treatment in a model study demonstrated site-specific delivery of the conjugate, reduced ischemic area, and reduced ROS levels compared with free drug combination [99]. Similarly, platelet membrane-cloaked polymeric nanoparticles with tPA (PNP-tPA) resulted in targeting and local clot degradation, with lower bleeding risk than free t-PA, improving the 20-day survival from 20% with tPA to 70% with PNP-tPA [100]. Liposomes with neuroprotectant (tacrolimus) significantly suppressed neuronal damage and ameliorated motor function deficits at one week of the middle cerebral artery occlusion (MCAO) rats [101].

#### 2.2.4. Nanoparticles with Targeting Ligands

To improve the specificity and efficacy of the thrombolytic agent, nanoparticles are conjugated with a specific ligand targeting fibrin, activated platelets, or factor XIII, which are associated with thrombus formation [102]. Fucoidan has a strong affinity for P-selectin expressed by the activated platelets during thrombus formation, hence nanoparticles with it have been shown to result in targeted delivery by tracking the platelet density and have shown improved thrombolysis efficacy (thrombus density reductions 29.5% vs. 59% with tPA) [103]. Other examples include L-carnosine peptide conjugated magnetic nanoparticles (LMNP) loaded with dexamethasone (dm@LMNP), which showed their effective BBB crossing with the potential application in ischemic stroke [104]. Magnetic nano vesicles (MNV) derived from iron oxide nanoparticles (IONP)-harboring MSC possess the advantage of magnetic navigation, which increased the localization to the ischemic lesion by 5.1 times [105].

## 3. Nanoconjugates to Protect the Brain from I/R Injury

In stroke, protecting cerebral vasculature is an important target to prevent the breakdown of the BBB, which could lead to inflammatory cells migrating to the brain parenchyma, oxidative stress, and edema that can further trigger a downstream secondary reperfusion injury (Figure 3). Even if the clot is successfully resolved, reperfusion injury still entails a pro-inflammatory cascade akin to ischemic tissue changes [106]. Besides the reperfusion injury, hemorrhagic conversion of ischemic stroke represents another possible early sequela, either as a spontaneous event or after recanalizing therapy use. In such instances, subsequent pathophysiology is shared with the course of parenchymal hemorrhagic stroke. Therefore, the development of effective combination therapy for stroke that provides not only thrombolysis but also attenuates secondary I/R injury is also necessary.

### 3.1. Nanoparticles with Antioxidant Agents

Metal nanoparticles acting as free radical scavengers such as cerium, platinum, selenium, and gold nanoparticles have shown protective effects in animal I/R injury models by regaining redox balance and preserving mitochondrial integrity [107,108]. Experiments also included the use of a targeting ligand with cerium nanoparticles such as integrin αvβ3, which is upregulated during ischemic injury [109]. These conjugated nanoparticles demonstrated better outcomes in terms of improved neurologic impairment scores, decreased infarction volume, decreased BBB disruption, oxidative stress, and neuronal apoptosis in the MCAO rat model [110]. Additional efforts included combining cerium nanoparticles with edaravone, another antioxidant, for a synergistic effect in neutralizing ROS [111]. Natural antioxidants such as resveratrol, a polyphenol antioxidant [112] resveratrol conjugated to low-density lipoprotein receptor [113], curcumin [114], nanozyme [115], melanin nanoparticle [115], melanin nanoparticles combined with MSC [116], betulinic acid (BA) [117], and glyburide-loaded BA [118] have been produced a protective effect in various models assessing their effectiveness against I/R injury-related oxidative stress.

In our efforts to address the issue of I/R injury, we encapsulated antioxidant enzymes, either catalase (nano-CAT) or superoxide dismutase (nano-SOD) or a combination of both (nano-SOD/CAT) in biodegradable, PLGA-based nanoparticles. We tested them in vitro, both in neurons and astrocytes, for their protective effect against hydrogen peroxide-induced oxidative stress [119,120]. The subsequent studies were carried out in the MCAO rat model of I/R injury [121] and in a thromboembolic model [122]. The treatments were given via the carotid artery at the time of reperfusion. In the thromboembolic model, it was a sequential treatment, tPA, followed by nano-SOD/CAT. The advantage of using antioxidant enzymes is their catalytic mechanism of action; hence they are very effective in neutralizing ROS even at low doses [123]. The compounds with antioxidant properties are inactivated after interaction with ROS, necessitating repeated dosing to maintain their therapeutic levels, which could be challenging in a clinical setting.

**Figure 6:**
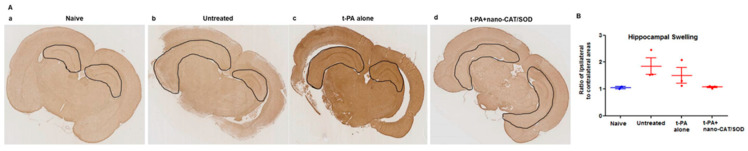
Immunohistochemical analysis of brain sections for hippocampus swelling. Following thromboembolic stroke, animals received different treatments at 3 h, and brains were collected at 48 h post-stroke. Brain sections were stained for GFAP. (**A**) Animals treated with tPA + nano-CAT/SOD show reduced swelling in the hippocampus than untreated and tPA alone treated animals. (**B**) Ratio of the ipsilateral to contralateral hippocampal areas showed reduced hippocampal swelling in tPA + CAT/SOD treated than in other groups. Data as mean ± s.e.m, naïve = 2, other groups = 3 animals in each group. *p* = 0.06 for untreated vs. tPA + nano-CAT/SOD treated; *p* = 0.13 for tPA alone vs. tPA + nano-CAT/SOD treated. Reproduced with permission from [122], copyright 2016 Elsevier.

Our data demonstrated the protective effect of nano-SOD and nano-CAT in neurons [119] and astrocytes [120] from oxidative stress in cell culture experiments. In the MCAO rat model, many aspects of I/R injury were inhibited following treatment with enzyme-loaded nanoparticles, which had favorable effects including protection of the BBB, inhibition of edema, reduction in infarct volume, improved neurological recovery, and long-term survival [121]. Interestingly in the embolic stroke model, tPA-only treatment was seen to inhibit the migration of progenitor cells and stem cells from the subventricular zone, but the combination treatment, i.e., tPA followed by nano-CAT/SOD, restored the above cellular activities, promoting neurogenesis [122]. In the above study, the sequential treatment of tPA followed by nano-CAT/SOD inhibited edema (Figure 6), indicating protection of BBB from I/R injury [122]. Since our initial studies, another group has shown similar efficacy with antioxidant enzyme-loaded nanoparticles [124]. Recent efforts include developing ROS-targeting nanomedicines containing an antioxidant agent to achieve target-specific delivery to the ischemic tissue [125]. Nanozyme, a polymer complex of antioxidant enzymes, has similarly shown the protective effect from I/R injury, including protecting neurons and the cerebral vasculature [126,127].

### 3.2. Nanoparticles with Anti-Inflammatory Agents

To overcome post-ischemic neuro-inflammatory damage, various anti-inflammatory conjugates have been studied in stroke models. For example, the liposomal cyclosporine A [128] and berberine nano micelles [129] have been reported to reduce microglial immunoreactivity, inflammatory responses, and infarct lesion volumes. Nanoparticles conjugated with monocyte membrane and rapamycin have been shown to inhibit the proliferation of neutrophils [130], reversing the pattern of inflammatory cytokines expression, resulting in improved neurological function [131]. In a few instances, the compound has pleiotropic effects. For example, bioactive nanoparticle engineered from a pharmacologically active oligosaccharide material (termed as TPCD), prepared by covalently conjugating a radical-scavenging compound (Tempol) and phenylboronic acid pinacol ester (PBAP) on β-cyclodextrin, decreased infarct volume and accelerated recovery of neurological function in the MCAO mice model, which was achieved by its simultaneous antioxidative, anti-inflammatory, and antiapoptotic effects [132].

## 4. Mechanical and Biological Stimulus

To loosen the clot or to improve the efficacy of thrombolytic agents, external mechanical stimuli such as ultrasound (sonothombolysis) [133,134], or external magnetic field with formulations containing magnetic nanoparticles carrying thrombolytic agents, have been explored. It is feasible to guide magnetic nanoparticles to the occlusion site using an external magnetic field to enhance the transport of tPA to the thrombus and increase the tPA-mediated thrombolysis [135,136,137]. The use of thrombus-targeting liposomal nanobubbles with concurrent high-intensity ultrasound (4.0 W/cm^2^) in a rabbit model of iliofemoral artery thrombosis showed recanalization in 90% of the animals as compared to 40% with tPA, or 20% with platelet non-specific nanobubbles and the same intensity of ultrasound [133]. Clinical trials with microbubbles and ultrasound increased the rate of recanalization and better outcomes in younger patients but with an increased risk of hemorrhagic complications or brain edema [138,139]. In another study, thrombus-targeting magnetic nanoparticles were developed by functionalizing iron oxide nanoparticles with an antibody recognizing activated integrin αIIbβ3. In this case, magnetic hyperthermia made the clot susceptible to the tPA-mediated thrombolysis [140].

Shear-activated nanoparticles taking the advantage of the distortion of the bloodstream caused by the clot itself, or aggregating nanoparticles releasing the tPA at the site of the stenosis, successfully recanalized blood vessels in the mouse model of embolism [141]. A similar approach has been explored by using a temporary endovascular bypass with a shear-activated nanotherapeutic that releases tPA when exposed to high levels of hemodynamic stress at sites of partial vascular occlusion in a rabbit model [142]. Anti-fibrin antibody targeting is one of the most effective methods for efficiently delivering tPA to the thrombus. Anti-fibrin antibodies deliver t-PA to the thrombus site in an inactive state, subsequently triggering its controlled activation, thereby reducing the risk of bleeding [143]. Zhang et al. have developed a self-assembling nanoformulation containing a photothermal sensitizer and a photothermal-activable nitric oxide donor. Following laser exposure, nanoformulation generated heat that facilitated its penetration into the clot and also generated nitric oxide. In animal models of acute ischemic stroke, the nanoformulation demonstrated better thrombolysis than the nanoparticle formulation without the photoactivation feature, and the produced nitric oxide prevented the recurrence of the clot formation [144].

## 5. Interactions at the BBB

Blood brain barrier integrity is compromised in the setting of acute stroke, but also following application of standard recanalizing therapies [145]. Mechanical thrombectomy causes local physical injury [146], while thrombolytics induce physiological changes which make the BBB more permeable [147]. Even in case of successful recanalization, I/R injury contributes to a variable degree of BBB compromise [148]. All these factors should be considered when investigating the application of nanoparticles to ameliorate some of the changes caused by ischemia as the primary event and associated secondary processes. Modifying nanoparticles by decreasing their size, maintaining a net positive charge, or by conjugating with ligands which are transcytosis transporter substrates, enhances the transport across an intact BBB [149]. By conjugation with cell-penetrating ligand, nanoparticles bypass the transcytosis pathway, while a formulation enabling a prolonged half-life further enhances the overall delivery to the target tissue, i.e., movement across BBB [149]. In the setting of acute injury which compromises the integrity of BBB, nanoparticles are able to penetrate the injured tissue site more easily [150]. The latter has been shown to occur in the setting of metabolic insults such as hypoxia [151], but experimental BBB disruption with osmotic, mechanical (ultrasound), or magnetic stimulus leading to greater NP penetrance has also been demonstrated [152]. In a clinical setting, this could be translated as therapeutic use of ultrasound to temporarily increase BBB permeability [153], while the NP-based therapy would be administered.

## 6. Clinical Application

Although nanotechnology is currently not approved for treatment of acute stroke or for neurorehabilitation, a considerable number of in vivo animal model studies and limited human studies offer hope and opportunities for translation into actual tools for human clinical practice (Table 1). There are multiple experiments which show how nanotechnological compounds could address stroke-associated pathophysiological processes (Table 1).

Nanoparticles with paramagnetic properties have been used as conjugates to target specific proteins in acute stroke as well as atherosclerosis. Such nanoconjugates can target intracellular or extracellular targets. Initial studies showed that smaller particles such as ultrasmall superparamagnetic iron oxide nanoparticles (USPION), with a size of less than 50 nm, have a longer half-life than larger ones, yet contrast labeling is superior with larger particles [154]. In a proof-of-concept study involving 11 subjects scheduled for carotid endarterectomy [155]. Kooi et al. reported that administration of USPION was associated with subsequent nanoparticle intake in high-risk or ruptured plaque, which was visualized with MRI and later confirmed by histopathology. In the ATHEROMA study [156], Ferumoxtran-10 as a USPION was successfully used to assess plaque status following statin use as part of the observed intervention. To boost the quality as contrast enhancers while also maximizing their additional clinical potential, micro-sized paramagnetic iron oxide nanoparticles (MPION) conjugated with antibodies aimed at receptors associated with endothelial activation, a process occurring in acute stroke, have been developed. Conjugates of such size, at the verge of nano- and microtechnology, were able to bind to activated endothelial surface proteins, e.g., vascular cell adhesion molecule-1 (VCAM-1), and act as adequately specific and sensitive contrast agents [157]. In a similar fashion, a MPION conjugate targeting P-selectin [158], or a different one targeting intercellular adhesion molecule-1 (ICAM-1) [159], were used to detect activated endothelium. This could help detect different phases of stroke, even transient ischemic attack (TIA)-related pathophysiology. Citicoline, a substance with neuroprotective effects, was combined with an immunoliposome targeting VCAM-1 [160]. It was successfully used as a theragnostic agent when the final nanoparticle reached the activated endothelium and concurrently represented a signal on chemical exchange saturation transfer (CEST) MRI. The presented capabilities would be invaluable for establishing stroke etiology, assessment of vascular inflammation and tissue at risk, or guiding further diagnostics and treatment. Moreover, nanoconjugates could also represent efficacy biomarkers when studying new or established stroke therapies.

Safety remains as a concern when considering nanoparticles for use in human subjects. Ideally, nanoconjugates should be biodegradable and non-toxic. Their profile should be favorable in terms of risks and benefits, and they indeed would represent a superior diagnostic or therapeutic modality as compared to the current standard. Targets identified in animal studies do not necessarily translate equally in the setting of the human body environment. The use of iron containing nanoparticles in acute inflammatory state may be limited by the possibility of local oxidative reaction following cellular intake. Therefore, preferred clearance could favor renal route or by enzymatic cleavage and phagocytic or endocytic mechanisms with ultimate non-toxic metabolism.

**Table 1:** Nanoparticles and nanoconjugates investigated for potential applications in ischemic stroke diagnosis, treatment, and study of cerebrovascular pathophysiology.

Nanoparticle or Nanoconjugate Used	Study Subjects	Objective	Reference
Ultrasmall superparamagnetic iron oxide particles	Human	Detection of high-risk or ruptured carotid artery plaque	[155]
	Human	Assessment of carotid plaque status following intervention (statin use)	[156]
Iron oxide microparticle conjugated with specific antibodies (P-selectin, ICAM-1)	Mouse	Detection of abnormal cerebrovascular endothelium due to transient ischemia	[158]
	Mouse	Detection of abnormal endothelium and migrating leukocytes in the setting of cerebrovascular injury (infarct, reperfusion)	[159]
Iron oxide nanoparticles with an antibody recognizing activated integrin αIIbβ3	Ex-vivo study (human blood clot)	Improved thrombolytic binding and affinity	[140]
Liposomal citicoline conjugated with VCAM-1	Mouse	Use of conjugate as a theragnostic agent	[160]
Liposomal tacrolimus	Rat	Use as neuroprotectant to reduce inflammation	[101]
Liposomal cyclosporine A	Rat	Reduction in infarct lesion size and inflammatory activity	[128]
Berberine nano micelles	Rat		[129]
PLGA-based nanoparticles with nano-catalase or superoxide dismutase	Mouse	Decrease in I/R injury with reduction in infarct volume, protection of the BBB integrity, and improved recovery	[121]
	Mouse	Decrease in I/R injury and amelioration of thrombolysis related decreased migration of subventricular zone stem cells	[122]
Cerium, platinum, selenium, or gold nanoparticles	Rat	Improving redox imbalance and preserving mitochondria	[107]
			[108]
Cerium nanoparticles with edaravone	Rat	Decreased oxidative injury	[111]
Resveratrol conjugated to LDL receptor	Ex vivo study (rat cell cultures)	Reduction in I/R related injury	[113]
Melanin loaded nanoparticles	Mouse		[116]
Glyburide-loaded betulinic acid nanoparticles	Rat		[117]
Encapsulated or conjugated tPA; PEGylated liposomes, silica-coated or gold nanoparticles, polymeric nano-constructs	Rat	Thrombolytic stability to enhance drug delivery to the target	[81]
	Mouse		[82]
			[85]
	In vitro and mouse		[86]
Nanoparticles conjugated with monocyte membrane and rapamycin	Rat	Reduction in neutrophil proliferation	[130]
Lipid nanoparticle conjugated with siRNA against Toll-like receptor-4	Mouse	Reduction in inflammation-induced microglial activation	[131]
Nanoparticle TPCD (Tempol with phenyloboronic acid pinacol ester and β-cyclodextrin)	Mouse	Decrease in infarct volume and acceleration of recovery	[132]
Nanoplatelet conjugated with neuroprotectant (ZL006e)	Rat	Reduction in infarct area and improved drug delivery across BBB	[99]
Platelet membrane-cloaked polymeric nanoparticles with tPA	Mouse	Improvement of thrombolytic profile with less hemorrhagic complications	[100]
MnCO_3_@BSA-ICG nanoconjugate	Mouse	Infarct area detection	[90]
NanoGd conjugate	Cynomolgus macaques	BBB permeability assessment	[93]
MSC-derived exosome nanovesicles combined with gold nanoparticles	Mouse	Targeted delivery to injured tissue	[97]
Nanoparticles with fucoidan	Mouse	Infarct area detection, improved thrombolysis	[103]
L-carnosine peptide conjugated magnetic nanoparticles loaded with dexamethasone	In vitro	Delivery of dexamethasone across BBB model	[104]

Abbreviations: BBB—blood brain barrier; BSA-ICG—bovine serum albumin and indocyanine green; Gd—gadolinium; I/R—ischemia/reperfusion; ICAM-1—intercellular adhesion molecule-1; LDL—low density lipoprotein; MSC—mesenchymal stem cells; PEG—polyethylene glycol; PLGA—poly (lactic-co-glycolic acid); siRNA—small interfering ribonucleic acid; tPA—tissue plasminogen activator; VCAM—vascular cell molecule-1.

## 7. Concluding Remarks

Nanotechnology offers multiple opportunities, from improving thrombolysis to adding complementary features such as imaging and/or neuro/vasoprotection from inflammation and reperfusion injury. The versatility of nanotechnology, with the ability to deliver different types of therapeutics or modify the surface with targeting ligands and the ability to formulate with different materials and compositions, allows one to design and develop multifunctional nanoparticles to address different aspects of stroke diagnostics and treatment. The most critical issue at this point is extending the time window for treatment and achieving effective recanalization. Another important aspect is making the thrombolytic agent safe, which will expand its clinical use, as the technology is now underutilized due to safety concerns. It also must be recognized that most preclinical studies are carried out in otherwise healthy animals, and patients suffering from stroke may have reduced cerebrovascular reserve or reduced benefits from protective agents due to underlying pathologies or aging.
